# Assessing Social Drivers of Health Among Pediatric Solid Organ Transplant Patients Using Psychosocial Screening: Rates of Risk and What DO we DO?

**DOI:** 10.1111/petr.70311

**Published:** 2026-04-03

**Authors:** Kelly E. Rea, Catherine Dusing, Kathleen G. Fredericks, Sriram Swaminathan, Melissa K. Cousino, Emily M. Fredericks

**Affiliations:** ^1^ Division of Pediatric Psychology, Department of Pediatrics C. S. Mott Children's Hospital, Michigan Medicine Ann Arbor MI USA

**Keywords:** clinical screening program, psychosocial risk, social drivers of health

## Abstract

**Background:**

Disparities in pediatric solid organ transplantation (SOT) outcomes persist despite increasing awareness. Efforts to reduce such disparities may include increasing screening to identify and mitigate social drivers of health (SDOH); however, research is needed to understand current clinical screening efforts and their impacts on referral outcomes.

**Methods:**

Caregivers (*N* = 302) of pediatric patients (0–20 years) completed the Psychosocial Assessment Tool (PAT 2.0) during annual multidisciplinary transplant clinic visits to identify SDOH and psychosocial risk. Total PAT scores were categorized into Universal, Targeted, or Clinical psychosocial risk ranges. Descriptive data from medical charts and PAT responses were analyzed to evaluate referral patterns, follow‐up rates, and adverse SDOH prevalence.

**Results:**

The majority of PAT scores were within the Universal risk range (65%), with heart transplant families reporting significantly higher psychosocial risk levels compared to liver and kidney (*F*(2, 298) = 5.22, *p* = 0.006). Most families (83%) identified at least one potentially adverse SDOH. Those screening Targeted or Clinical reported significantly greater average number of SDOH risk factors compared to Universal range (*F*(2, 299) = 38.75, *p* < 0.001). One year post‐screening, the majority of families in the Targeted or Clinical risk range completed follow‐up with at least one psychosocial service, including social work (86%), mental/behavioral health providers (41%), and educational specialist interventions (7%).

**Conclusions:**

Elevated rates of psychosocial risk in pediatric SOT continue to persist; therefore, standardized clinical assessments to identify potentially adverse SDOH or healthcare barriers are necessary to provide individualized support. Implementing routine screening with appropriate referrals is a crucial step in addressing SDOH disparities throughout the transplant process.

## Graphical abstract

The majority of families identified at least one potentially adverse SDoH and also followed up with some form of psychosocial services. Elevated psychosocial risk rates in pediatric solid organ transplantation reinforce the need for routine clinical assessments and appropriate referrals to address potentially adverse social drivers of health or barriers to care throughout the transplant process.

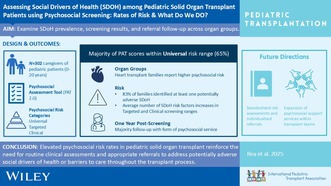

**Abbreviations**
PAT: psychosocial assessment tool
SDOH: social drivers of health
SOT: solid organ transplant

Solid organ transplantation (SOT) is a common treatment for end‐stage kidney, liver, or heart failure among pediatric patients. However, disparities across the transplant process, including pre‐transplant evaluation, listing, and post‐transplant outcomes, persist, most commonly related to racial or ethnic identity [1]. Notably, the presence of disparities associated with commonly assessed metrics in medicine, such as racial or ethnic identity and socioeconomic or insurance status, are driven by mechanisms within and beyond healthcare systems, including but not limited to systemic racism within healthcare systems and society at large, individuals' mistrust of health systems, and socioeconomic barriers to engagement with healthcare systems and health promoting activities at the community level [1]. As such, these require a comprehensive understanding and systems‐level change to address [1]. Social drivers of health (SDOH) are non‐medical factors and conditions that influence health outcomes, including the systems and policies that shape daily life and interactions with health care service and delivery [2]. Healthy People 2030 details a conceptual framework describing SDOH across several domains, including Education Access and Quality, Health Care and Quality, Neighborhood and Built Environment, Social and Community Context, and Economic Stability [3]. Each of these domains interacts with and impacts the others as it relates to an individual's health. A comprehensive understanding of the ways in which these domains are present in the lives of pediatric SOT recipients and their families, as well as the individualized ways healthcare providers may be able to identify and mitigate the impacts of SDOH on health outcomes, is essential [1, 4, 5, 6].

Providers can identify and address concerns in the healthcare setting through the use of screening efforts. The importance of clinical screening programs in pediatric SOT with connection to appropriate resources has been established in other critical domains, such as health and behavioral screening in pre‐transplant evaluations [7, 8], and universal post‐transplant neuropsychological screening [9]. Despite these recommendations, barriers to implementation of screening programs in the multidisciplinary clinic setting may include added time burden for families, busy clinic visits involving multiple providers, billing requirements, lack of mental health insurance coverage, and limited availability of psychosocial providers to meet with patients or complete screening tools. Some of these barriers may be alleviated, in part, by combining assessment of salient factors warranting use of screening measures, such as SDOH screening, with general psychosocial risk assessment. However, limited examples of this exist in the literature to date.

Further, examination of SDOH among pediatric SOT recipients and families is often limited by the existing literature's focus on a select few, often unmodifiable, SDOH, such as caregivers' or patients' racial or ethnic identity [1]. Given the multifactorial, intersecting impact of SDOH, use of a systematic screening effort across several domains is important to best understand how to support families. A recent systematic review of broad SDOH screeners across children noted the importance of SDOH screening across domains, followed immediately by referrals and interventions aimed at assessing identified concerns or barriers to care [10]. However, results also highlighted unknowns regarding the effectiveness of these referrals or the provision of resources in meeting identified needs beyond the time of screening [10]. Given the medical complexity associated with managing a transplanted organ and the noted impacts of SDOH on health outcomes, including rejection and graft loss [1, 4, 5, 6], it is critical that we begin to outline best practices in screening and addressing such drivers of health.

The present study represents efforts of a clinical psychosocial screening program implemented pragmatically within a single‐center institution across pediatric heart, liver, and kidney SOT programs. Study aims included (1) describing the prevalence of SDOH across pediatric SOT groups through the established psychosocial screening program and (2) examining potential differences in SDOH and psychosocial screening results across organ groups. Finally, the present study aimed to (3) describe outcomes of the clinical screening program, including screening results, provision of referrals following screening, and follow‐up with referrals within 1 year of screening as a means of addressing SDOH and barriers to care.

## 1 Method

### 1.1 Participants

Participants included caregivers of pediatric SOT recipients (ages 0–20 years) who were at least 6 months post‐transplant and seen in a multidisciplinary heart, liver, or kidney transplant clinic setting at a Midwest academic medical center. Contextualizing the volume of the current transplant center, from 2014 to 2023 (the duration of the current psychosocial screening initiative), 352 pediatric organ transplants occurred across heart, liver, and kidney programs. Exclusion criteria for the present study included caregivers who were not fluent in English or Spanish, given the language availability of the administered survey, or pediatric patients who attended their transplant clinic visit with someone other than a parent or guardian.

### 1.2 Procedures

The current project is part of a broader quality improvement clinical effort and is IRB exempt, deemed not regulated (HUM00082618). Outpatient transplant clinics included various psychosocial team members, including Transplant Psychologists (Heart, Kidney, Liver; supporting mental health, coping, adherence, etc.), Social Workers (Heart, Kidney, Liver; supporting connection to resources, travel coordination, insurance, guardianship, etc.), and dedicated Education Specialists (Heart; bridging medical setting and school community, supporting 504 and Individualized Education Plans [IEPs], etc). The current psychosocial screening clinical initiative began in the pediatric liver program in 2014 and in the pediatric heart and kidney programs in 2016. As this was a clinical, quality improvement‐based program, efforts were made to administer the psychosocial screening survey annually to eligible patients/families; however, the availability of psychosocial team members, patient/family medical status, the COVID‐19 pandemic, and other factors sometimes prevented annual screening. Screening was administered by psychosocial team members via paper and pencil survey. One caregiver from each family was invited to complete the Psychosocial Assessment Tool (PAT 2.0) [11] on behalf of their family system during the clinic visit on an annual basis to inform psychosocial risk and inform allocation of resources. In the instance of multiple available PAT screenings from a single family, the most recent completion was used in the present study.

### 1.3 Measures

Psychosocial Assessment Tool (PAT 2.0). The PAT 2.0 [11] is a brief caregiver‐reported psychosocial screening tool to assess family system psychosocial risk, which has been widely validated across pediatric medical populations. The PAT is based on the Pediatric Psychosocial Preventative Health Model (PPPHM) framework [12], which categorizes levels of psychosocial risk into Universal, Targeted, or Clinical based on the total PAT score (total score ranges from 0 to 3). The PAT includes 7 subscales: Family Structure and Resources, Social Support, Child Problems, Sibling Problems, Family Problems, Parent Stress Reactions, and Family Beliefs. Additional demographic information is also collected. PAT Total score is calculated by summing subscale scores and ranges from 0 to 7. A PAT Total score of < 1 is the lowest risk category (Universal Risk) and indicates few patient and/or family problems and stressors [11]. A PAT Total score between 1 and 1.99 (Targeted Risk) indicates some patient and/or family problems and stressors. A PAT Total score of > 2 is the highest risk category (Clinical Risk) and indicates many patient and/or family problems and stressors [11]. Risk categorizations inform the level of recommended interventions, including continued universal levels of screening (Universal Risk), closer monitoring of risk factors or consideration of interventions (Targeted Risk), or referral for more intensive psychosocial services (Clinical Risk). The PAT 2.0 has been validated for use in pediatric SOT [13, 14].

PAT surveys were hand‐scored at the time of clinical encounter by psychosocial providers (e.g., Social Workers, Psychologists, Psychology trainees) and entered into patients' medical charts with clinical encounter documentation. Item‐level data and scores were subsequently entered into secure, HIPAA‐compliant REDCap (Research Electronic Data Capture) data storage by trained research staff at the University of Michigan Health System [15, 16]. REDCap is a secure, web‐based software platform designed to support data capture for research studies, providing (1) an intuitive interface for validated data capture; (2) audit trails for tracking data manipulation and export procedures; (3) automated export procedures for seamless data downloads to common statistical packages; and (4) procedures for data integration and interoperability with external sources.

In the current investigation, item‐level responses of the PAT were further assessed to gain an understanding of the prevalence of SDOH among families of children who underwent SOT. PAT items of interest were selected based on conceptual overlap with the CDC Healthy People 2030 SDOH framework, such as adverse caregiver or childhood experiences, family financial instability, and access to transportation [17]. Initial review of items was completed by three authors and subsequently reviewed by co‐authors. Thirteen items were identified that corresponded to established CDC SDOH domains (i.e., Education Access and Quality, Health Care and Quality, Neighborhood and Built Environment, Social and Community Context, and Economic Stability).

### 1.4 Data and Analytic Plan

Medical Chart Review. To determine psychosocial referral and follow‐up patterns in response to psychosocial screening efforts, a medical chart review was undertaken for those families who screened in the elevated psychosocial range (Targeted or Clinical Total PAT scores). A data extraction template and criteria were established by the authorship group to assess referrals and recommendations at screening encounter (i.e., recommendation or referral to Social Work, mental/behavioral health, and developmental supports, or Education Specialist at the time of PAT screening). Referral/recommendation for each psychosocial service was coded as ‘Yes’ or ‘No.’ Similarly, the chart review examined whether subsequent follow‐up occurred with Social Work, mental/behavioral health, or developmental supports, or an Education Specialist within one year of the PAT screening encounter. Follow‐up with mental/behavioral health or developmental supports internal to the hospital system (as indicated by chart review), as well as external local services (as reported by patients or caregivers; e.g., patient following with local therapist, state‐based early intervention services, etc.) were coded as ‘Yes’ for follow‐up occurring.

For reliability purposes and data adjudication, one reviewer screened all charts while a second reviewer independently screened a random 20% of charts for reliability as determined by a random number generator. Review efforts achieved adequate reliability (i.e., > 80% agreement [18]). Discrepancies were discussed, and consensus was reached within the broader co‐author group.

Analyses. Data were extracted from REDCap and analyzed using SPSS version 27 [19]. Frequencies and descriptives were conducted as appropriate for item‐level response analyses and PAT Total scores and risk categorizations. One‐way ANOVA analyses were conducted to assess differences in PAT Total scores and frequency of potentially adverse SDOH endorsed across organ groups, with *p* < 0.05 considered statistically significant. In instances of dual‐organ transplantation (e.g., combined heart‐kidney), participants were excluded from analyses by organ group given overlapping group membership. Post hoc independent samples *t*‐tests were conducted to assess for differences in rates of psychosocial risk and referral patterns across pre‐ and post‐COVID‐19 time periods.

## 2 Results

### 2.1 Participant Characteristics

Participants included 302 caregivers of children and young adults ages 0 to 20 years (*M* = 9.28, SD = 4.94) who received heart (*n* = 77), kidney (*n* = 72), liver (*n* = 152), or combined heart/kidney (*n* = 1) transplants who completed psychosocial screening from October 2014 to October 2023. Full family demographics are presented in Table 1. Please see online Table S1 for the demographics breakdown by organ group. Based on self‐identification via survey responses, caregivers were predominantly married or partnered (60%), biological parents (91%), over age 21 (92%), of children who were White (72%) and non‐Hispanic. Fifty‐four percent of children were reported by caregivers as male.

**TABLE 1:** Family and patient demographics.

*N* = 302 caregivers	Mean (SD)/*n* (%)	Range
Transplant type		
Heart	77 (25.5%)	
Kidney	72 (23.8%)	
Liver	152 (50.3%)	
Combined heart/kidney	1 (0.3%)	
Caregiver role		
Biological parent	275 (91.1%)	
Adoptive parent	11 (3.6%)	
Grandparent	8 (2.6%)	
Legal guardian	4 (1.3%)	
Other/not reported	4 (1.3%)	
Caregiver(s) age		
Under age 21	3 (1%)	
Age 21 or over	278 (92%)	
One caregiver 21+, other < 21	12 (4%)	
Not reported	9 (3%)	
Caregiver education		
Started school but did not finish	30 (9.9%)	
Finished high school/got GED	83 (27.5%)	
Started college or trade school	57 (18.9%)	
Finished college or trade school	106 (35.1%)	
Started masters or doctoral program	11 (3.6%)	
Finished masters or doctoral program	41 (13.6%)	
Caregiver marital status		
Single	69 (22.8%)	
Married/partnered	180 (59.6%)	
Separated/Divorced	33 (10.9%)	
Other/not reported	20 (6.6%)	
Child age (years)	9.28 (4.94)	0–20 years
Child gender		
Female	137 (45.4%)	
Male	164 (54.3%)	
Not reported	1 (0.3%)	
Child race		
American Indian/Alaska Native	5 (1.7%)	
Asian	12 (4.0%)	
Black/African American	62 (20.5%)	
Hawaiian/Other Pacific Islander	1 (0.3%)	
Multiracial	10 (3.3%)	
White	216 (71.5%)	
Middle Eastern/Arabic	4 (1.4%)	
Other, not reported	1 (0.3%)	
Child ethnicity		
Hispanic/Latino	27 (8.9%)	
Not Hispanic/Latino	211 (69.9%)	
Not reported	64 (21.2%)	

### 2.2 Rates of Psychosocial Risk

**FIGURE 1:**
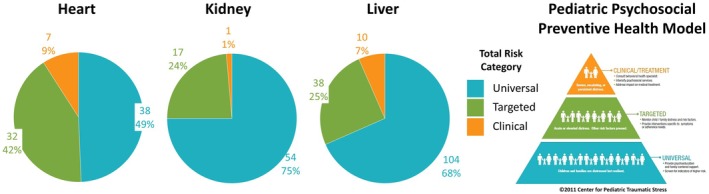
PAT Psychosocial Risk Category by Transplant Type. Source: PPPHM image copyright Center for Pediatric Traumatic Stress.

Across all families, caregivers reported that psychosocial risk was, on average, in the Universal range (*M* = 0.87, SD = 0.65). Sixty‐five percent of families (*n =* 196) were in the Universal risk range, followed by Targeted (*n* = 88; 29%) and Clinical (*n* = 18; 6%). Rates of overall psychosocial risk differed significantly by organ transplant type, such that caregivers of children who had received a heart transplant reported significantly greater average levels of psychosocial risk (*M* = 1.05, SD = 0.71) compared to liver (*M* = 0.85, SD = 0.64) and kidney (*M* = 0.71, SD = 0.57; *F* (2, 298) = 5.22, *p* = 0.006). This is further supported by higher rates of Targeted or Clinical risk indicated by caregivers of children with heart transplants (51%, *n* = 39) as compared to kidney (25%, *n =* 18) and liver (32%, *n* = 48; see Figure 1). In post hoc analyses examining rates of psychosocial risk in pre‐ and post‐COVID time periods among those identified as Targeted or Clinical risk, there were no differences in the mean total PAT scores across time periods.

### 2.3 Rates of Social Drivers of Health

**FIGURE 2:**
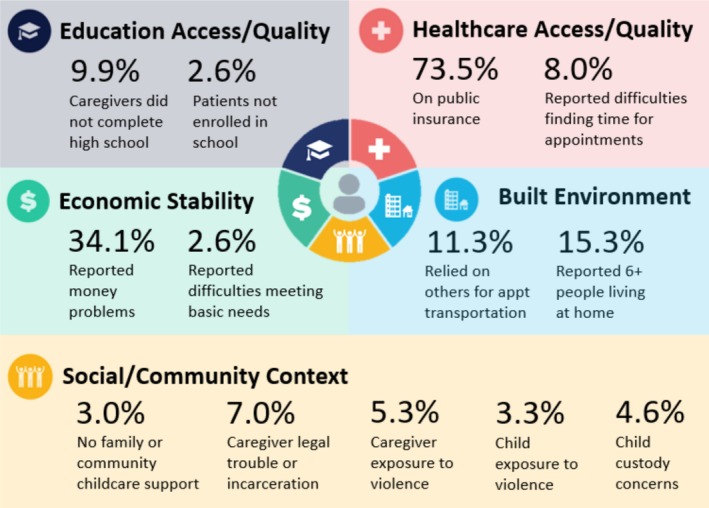
PAT Items by Healthy People 2030 SDoH Domains. Source: Image adapted from ODPHP Healthy People 2030. Healthy People 2030, U.S. Department of Health and Human Services, Office of Disease Prevention and Health Promotion. Retrieved May 7, 2025, from https://health.gov/healthypeople/objectives‐and‐data/social‐determinants‐health.

Over 4 in 5 families (83%) reported at least one potentially adverse SDOH. More than 2 in 5 families (41%) reported 3+ potentially adverse SDOH. On average, families endorsed 1.70 potentially adverse SDOH items on the PAT (SD = 1.27, range 0 to 6). This did not differ significantly by organ group (*F* (2, 298) = 0.52, *p* = 0.59). Those screening in the Targeted or Clinical risk ranges had a significantly greater number of potentially adverse SDOH items endorsed, *M* = 2.24 and *M* = 3.28, respectively, as compared to those in the Universal range (*M* = 1.31; *F* (2, 299) = 38.75, *p* < 0.001). Families screening Targeted or Clinical risk endorsed a greater number of SDOH items in the pre‐COVID data collection period as compared to post‐COVID, such that on average one additional SDOH item was endorsed in the pre‐COVID period as compared to post‐COVID, based on the mean number of SDOH items endorsed equal to 2.59 and 1.65, respectively. Aligned with Healthy People 2030 domains (See Figure 2), in the domain of *Economic Stability*, 34.1% of caregivers (*n* = 103) endorsed money problems, with 2.6% (*n* = 8) reporting difficulties meeting basic needs. Regarding *Education Access/Quality*, 9.9% of caregivers (*n* = 30) did not finish high school. Further, though no patients formally dropped out of school, 2.6% of school‐age patients (*n* = 8) were not currently receiving school or homebound services, per caregiver report. Regarding *Health Care Access/Quality*, 73.5% of patients had public insurance, and 8.0% of caregivers (*n* = 24) reported at least some difficulty finding time to attend children's appointments. Regarding *Neighborhood/Built Environment*, 11.3% of families relied on others for appointment transportation, and 15.3% reported ≥ 6 people living at home (*n* = 46). Regarding *Social/Community Context*, 3.0% of caregivers (*n* = 9) reported no family/community childcare supports, 7.0% (*n* = 21) endorsed legal trouble/incarceration, 4.6% (*n* = 14) endorsed child custody concerns, 5.3% (*n* = 16) endorsed caregiver exposure to crime/violence, and 3.3% (*n* = 10) endorsed child exposure to crime/violence. Please see online Supplementary Table 2 for the SDOH items breakdown by organ group.

### 2.4 Rates of Psychosocial Referrals/Follow‐Ups

Among families who screened Targeted or Clinical psychosocial risk across all organ groups (*n* = 106; 35%), 86% were seen by Social Work in the year following psychosocial screening (*n =* 91). Interventions included transportation assistance, parking and meal reimbursement, resource support, and caregiver/family check‐ins between medical visits. Further, 41% of families (*n* = 43) were seen by mental/behavioral health or developmental supports, including Transplant Psychology, state‐based early intervention services, physical, occupational, or speech therapies, or local community mental health providers. Lastly, 7% of families (*n* = 7) were seen by hospital‐based Education Specialists to support academic and educational transition planning, including support for 504 and Individualized Education Plans, acquisition of homebound services, and tutoring supports during inpatient admissions. Referrals and follow‐ups were individualized to meet specific patient and family needs, and follow‐up patterns were similar across organ groups, except for Education Specialist follow‐up, as there was only a dedicated Education Specialist available for the Heart Transplant clinic (see Table 2 for further details). In post hoc analyses examining referral rates among those identified as Targeted or Clinical risk in pre‐ and post‐COVID time periods, there were no significant differences in the number of referrals received after screening (i.e., services were still readily available pre‐ and post‐COVID).

**TABLE 2:** Psychosocial screening results and referral/follow‐up rates by organ group.

	Total (*N =* 302)	Heart (*n* = 77)	Kidney (*n* = 72)	Liver (*n* = 152)	*F, p*
Total PAT Score [*M, (SD)*]	0.87 (0.65)	1.05 (0.71)	0.71 (0.57)	0.85 (0.64)	5.22, 0.006**
Clinical Risk Category [*n* (%)]					
Universal	196 (65%)	38 (49%)	54 (75%)	104 (68%)	
Targeted	88 (29%)	32 (42%)	17 (24%)	38 (25%)	
Clinical	18 (6%)	7 (9%)	1 (1%)	10 (7%)	
Referral/Follow‐Up rates w/in 1 year of screening for Targeted/Clinical Risk [*n* (%)]					
Social Work	91 (86%)	38 (97%)	14 (78%)	38 (79%)	
Mental/Behavioral Health	43 (41%)	23 (59%)	6 (33%)	13 (27%)	
Education Specialist	7 (7%)	6 (15%)	1 (6%)	0 (0%)	
Number of potentially adverse SDoH items endorsed on PAT [*M, (SD)*]	1.70 (1.27)	1.65 (1.10)	1.60 (1.27)	1.77 (1.35)	0.52, 0.59

*Note:* ** *p* < 0.01.

## 3 Discussion

The current study sought to report the effects of a clinical psychosocial screening program and to describe the prevalence of SDOH across pediatric SOT recipients, explore differences in SDOH and psychosocial screening across organ groups, and examine outcomes of screening results. Most families across organ groups were in the Universal psychosocial risk category. When comparing organ groups, families of children who had received heart transplants indicated significantly higher average levels of psychosocial risk and higher rates of Targeted or Clinical risk categorization when compared to families of children who had received liver or kidney transplantation. Across organ groups, over 80% of families reported at least one potentially adverse SDOH, and over 40% reported three or more, and this did not differ by organ groups. In response to screening efforts, families who screened at Targeted or Clinical psychosocial risk levels were referred at high rates to available psychosocial services at the current institution based on individualized identified needs, with referral and follow‐up rates with at least one psychosocial service occurring for 86% of families.

When compared to other pediatric illness groups, rates of psychosocial risk across organ groups in the present study were similar to those of families with recent cancer diagnosis [20], kidney transplant [14], and inflammatory bowel disease diagnoses [21]. Notably, within our own center, prior examination of psychosocial screening within heart transplant compared to the current investigation indicates increased average total psychosocial risk scores from the Universal to Targeted range (0.96 vs. 1.05) and higher rates of families with heart transplants falling in the Targeted or Clinical risk range (41% vs. 51%) over time [13]. These differences in psychosocial risk across organ groups and over time are likely multifactorial. Families awaiting heart transplantation may require more frequent hospital admissions or await heart transplantation in the hospital due to critical illness or need for other advanced cardiac therapies during the waitlist period (e.g., ventricular assist device). Such experiences may negatively impact patients’ and caregivers’ functioning due to impacts on work, finances, and family systems more broadly. Further, among pediatric recipients, heart transplantation is associated with a higher risk of mortality at one, five, and 10‐year post‐transplant follow‐up [22], which may further impact patient and family coping in the post‐transplant period [23]. Additionally, given the potential for acute decompensation of pediatric patients' medical status, indicating the need for heart transplantation, assessing and addressing potential psychosocial risk factors may occur primarily after transplantation, rather than preemptively. Those awaiting kidney or liver transplantation may be able to be appropriately compensated on dialysis or receive living‐related donor organs, limiting the reliance on lengthy waitlist times and allowing for additional psychosocial intervention in the pre‐transplant period when warranted. Lastly, the COVID‐19 pandemic reshaped healthcare systems and illuminated disparate healthcare needs and access [24]. Such disruptions included increased mental health concerns [25] and contributed to disruption in family systems, work, and finances, and overall psychological stress. These impacts have been similarly reported among patients and families with congenital heart disease, with added stressors associated with medical complexity [26]. As SDOH continue to contribute to widening gaps in health and wellbeing across the population [27], there remains both growing patient and family need, as well as increased awareness and efforts to address these needs and disparities.

Prevalence of SDOH in the present pediatric organ transplantation sample is largely consistent with recent national United States data indicating persistent inequities in a number of SDOH indicators across the general population [28]. There remain significant disparities in health outcomes associated with access to health insurance, level of education, income, and employment across the United States [28]. Further, recent survey data highlight one in three adults in the US have limited health literacy knowledge or skills [29], with both parental and child health literacy associated with worse child health outcomes [30, 31]. This is notable when considering the current sample in which almost 10% of caregivers of pediatric organ transplant recipients did not complete high school and over one in three families reported having financial difficulties. Among pediatric patients, a recent study found those with both lower income and lengthy travel time to appointments by either car or bus were significantly less likely to attend clinic appointments in a single‐center study [32]. In the present study, over 1 in 10 families relied on others or public transportation to attend appointments, representing an additional potential barrier to access to care. Each of these SDOH domains represents critical areas for intervention and warrants additional support in the clinical setting following screening efforts.

In two investigations of psychosocial risk among heart and kidney transplant recipients, higher PAT total scores representing greater psychosocial risk were predictive of increased likelihood for referral or receipt of psychosocial services within two years of screening among kidney transplant recipients [14], and over 50% of those screening clinical risk for patient concerns specifically were referred for mental or behavioral health follow‐up among heart transplant recipients [13]. In the current study, approximately 9 in 10 families received at least one referral or completed follow‐up after psychosocial risk screening based on identified individual needs. These supports ranged from transportation assistance, parking, and meal reimbursement, resource or therapy referral lists, referral to internal Psychology or Neurodevelopmental services, and support related to educational intervention needs, such as 504 or Individualized Education Plans, or establishing homebound services. Notably, not all families received the same levels of support based on identified needs, and some subscales of the PAT may indicate additional risk but do not necessarily result in follow‐up given the availability of psychosocial services (e.g., sibling behavioral concerns). As such, it is important to consider the results of psychosocial screening in tandem with discussions with families about what services they may already be receiving, are interested in, and in the context of the availability of supports.

Current findings highlight several important clinical implications. First, the high prevalence of SDOH and elevated rates of psychosocial risk in this pediatric solid organ transplant sample support the need for standardized assessment in this clinical population. Without standardized assessment occurring across all patients and families, it is likely that the identification of risk factors or barriers to care will be underestimated and therefore under‐addressed. Further, it is not enough to merely screen and identify SDOH and psychosocial risk. It is critical to take steps to meet the identified needs of patients and families in an effort to reduce barriers and support health outcomes. Among pediatric solid organ transplant teams, this includes tangible, institutional support for dedicated psychosocial team members, including social workers, psychologists, psychiatrists, and education specialists. Additionally, advocacy at the institutional, state, and federal levels should include support for increasing access to health‐promoting care. This may include incorporating standardized SDOH screening into the electronic medical record and advocating for reimbursement models that reflect time spent on psychosocial evaluations and care coordination efforts and support access to mental health and behavioral interventions. Further, community and local organizations may present additional partnership opportunities to address housing and food insecurity and transportation access, as well as increasing telemedicine access to community mental health providers. Lastly, while pre‐transplant psychosocial evaluation is standard practice at the onset of the transplant process, it is important to continue assessment of SDOH and psychosocial risk post‐transplant, as risk factors may evolve over time [8].

Strengths of the current study must be considered in the context of limitations, which inform future directions. Findings represent a single center, with the sample being predominantly biological, partnered parents of White race, which limits generalizability to other centers and demographics. Future studies should explore the prevalence of SDOH and assess the impact of associated screening and referral programs among pediatric solid organ transplant recipients and families across greater geographical and sociodemographic diversity. Further, larger, multi‐site investigations would allow for greater statistical power to examine potential impacts of intersectionality across SDOH and salient outcomes related to psychosocial risk and rates of follow‐up. Additionally, while the current psychosocial assessment tool is widely used in pediatric medical settings, it does not represent a comprehensive assessment of SDOH. Using one assessment tool annually allows for decreased family burden in completing surveys during appointments. However, the PAT does not assess for the full scope of other potentially adverse SDOH, including experiences of systemic racism or discrimination, medical provider mistrust, access to nutritious food or clean water, or quality of housing. Use of a screening tool may prompt additional conversations, which allow for assessment of these and other concerns that may further impact children and families' health related to transplant. Future studies and clinical program efforts may warrant assessment of SDOH using dedicated screening tools (e.g., Structural Vulnerability Assessment Tool). Additional research may include exploring the merits of universally applied SDOH‐specific screening vs. targeted screening efforts when indicated based on preliminary broad psychosocial screening. Further, while every effort was made to capture families' screening results annually, some families may not have completed due to availability within a busy medical clinic appointment or psychosocial team member bandwidth, lack of interest in participating in the screening program, or language availability for the primary language spoken. In the current psychosocial screening program, the PAT was available in both English and Spanish. However, for large transplant centers serving a wide range of languages spoken, PAT translations are available for additional languages to increase access, including Bahasa (Indonesia), Brazilian Portuguese, Chichewa (Malawi), Chinese/Mandarin, Danish, Dutch, Estonian, Farsi, Finnish, French (Canada), Greek, Hebrew, Italian, Japanese, Polish, and Setswana [33]. Lastly, the current screening tool and program focused on caregiver reports of psychosocial risk; therefore, the patient perspective for those younger than 18 is not captured. While pediatric patients' understanding and ability to report on all potentially adverse SDOH may be more limited (e.g., family finances, awareness of health insurance information), assessing patients' own perspectives on potential psychosocial risks and barriers to care would add a layer of information for psychosocial teams and services.

The current study demonstrates both a high level of psychosocial need and rates of potentially adverse SDOH among pediatric organ transplant recipients and their families, as well as a model for implementation of a psychosocial screening program with individualized referrals to meet this growing need. Results support the continuation and expansion of psychosocial support services within multidisciplinary transplant teams. Dedicated team members to support patients and families in assessing and intervening upon identified psychosocial risk and potentially adverse SDOH are critical not only during pre‐transplant evaluations, but also in post‐transplant follow‐up to promote access to care, reduce barriers, and support better health outcomes.

## Funding

This work was supported by Dr. Ida Malian and the University of Michigan Transplant Center.

## Supporting information


**Supplementary Table 1** Family and patient demographics reported by organ group.
**Supplementary Table 2** PAT items by Healthy People 2030 SDOH domains reported by organ group.

## Data Availability

The data that support the findings of this study are available on request from the corresponding author. The data are not publicly available due to privacy or ethical restrictions.
