# Supplementary material for: Assessing Social Drivers of Health Among Pediatric Solid Organ Transplant Patients Using Psychosocial Screening: Rates of Risk and What DO we DO?
Source: Pediatr Transplant. 2026 Apr 3;30(4):e70311. doi: 10.1111/petr.70311 (PMC13047720; doi:10.1111/petr.70311)
Supplement: Supplementary file 1 — Supplementary Table 1 Family and patient demographics reported by organ group. Supplementary Table 2 PAT items by Healthy People 2030 SDOH domains reported by organ group. [file PETR-30-e70311-s001.docx]

Supplementary Table 1. Family and Patient Demographics by Organ Type

|  | Combined Organ Groups  (*N=*302 caregivers) | Heart  (*n* = 77 caregivers) | Kidney  (*n* = 72 caregivers) | Liver  (*n* = 152 caregivers) |
| --- | --- | --- | --- | --- |
|  | Mean (*SD*) / n (%) |  |  |  |
| Caregiver role |  |  |  |  |
| Biological parent | 275 (91.1%) | 71 (92.2%) | 65 (90.3%) | 138 (90.8%) |
| Adoptive parent | 11 (3.6%) | 1 (1.3%) | 3 (4.2%) | 7 (4.6%) |
| Grandparent | 8 (2.6%) | 2 (2.6%) | 1 (1.4%) | 5 (3.3%) |
| Legal guardian | 4 (1.3%) | 2 (2.6%) | 2 (2.8%) | -- |
| Other/not reported | 4 (1.3%) | 1 (1.3%) | 1 (1.4%) | 2 (1.4%) |
| Caregiver(s) age |  |  |  |  |
| Under age 21 | 3 (1%) | -- | -- | 3 (2.0%) |
| Age 21 or over | 278 (92%) | 74 (96.1%) | 63 (87.5%) | 140 (92.1%) |
| One caregiver 21+, other < 21 | 12 (4%) | 2 (2.6%) | 4 (5.6%) | 6 (3.9%) |
| Not reported | 9 (3%) | 1 (1.3%) | 5 (6.9%) | 3 (2.0%) |
| Caregiver education |  |  |  |  |
| Started school but did not finish | 30 (9.9%) | 7 (9.1%) | 6 (8.3%) | 17 (11.2%) |
| Finished high school/got GED | 83 (27.5%) | 15 (19.5%) | 20 (27.8%) | 48 (31.6%) |
| Started college or trade school | 57 (18.9%) | 21 (27.3%) | 8 (11.1%) | 28 (18.4%) |
| Finished college or trade school | 106 (35.1%) | 29 (37.7%) | 23 (31.9%) | 53 (34.9%) |
| Started post-graduate program | 11 (3.6%) | 4 (5.2%) | 4 (5.6%) | 3 (2.0%) |
| Finished post-graduate program | 41 (13.6%) | 14 (18.2%) | 9 (12.5%) | 18 (11.8%) |
| Caregiver marital status |  |  |  |  |
| Single | 69 (22.8%) | 22 (28.6%) | 13 (18.1%) | 34 (22.4%) |
| Married/partnered | 180 (59.6%) | 40 (51.9%) | 47 (65.3%) | 92 (60.5%) |
| Separated/Divorced | 33 (10.9%) | 9 (11.7%) | 6 (8.3%) | 18 (11.8%) |
| Other/not reported | 20 (6.6%) | 6 (7.8%) | 6 (8.3%) | 8 (5.3%) |
| Child age (years) | 9.28 (4.94) | 10.03 (5.14) | 9.94 (4.74) | 8.57 (4.87) |
| Child gender |  |  |  |  |
| Female | 137 (45.4%) | 38 (49.4%) | 31 (43.1%) | 68 (44.7%) |
| Male | 164 (54.3%) | 39 (50.6%) | 41 (56.9%) | 83 (54.6%) |
| Not reported | 1 (.3%) | -- | -- | 1 (.7%) |
| Child race |  |  |  |  |
| American Indian/Alaska Native | 5 (1.7%) | 3 (3.9%) | -- | 2 (1.3%) |
| Asian | 12 (4.0%) | 1 (1.3%) | 4 (5.6%) | 7 (4.6%) |
| Black/African American | 62 (20.5%) | 15 (19.5%) | 15 (20.8%) | 32 (21.2%) |
| Hawaiian/Other Pacific Islander | 1 (.3%) | -- | -- | 1 (.7%) |
| Multiracial | 10 (3.3%) | 4 (5.2%) | 1 (1.4%) | 5 (3.3%) |
| White | 216 (71.5%) | 61 (79.2%) | 50 (69.4%) | 104 (68.4%) |
| Middle Eastern/Arabic | 4 (1.4%) | -- | -- | 4 (2.6%) |
| Other, not reported | 1 (.3%) | -- | 1 (1.4%) | -- |
| Child ethnicity |  |  |  |  |
| Hispanic/Latino | 27 (8.9%) | 6 (7.8%) | 8 (11.1%) | 13 (8.6%) |
| Not Hispanic/Latino | 211 (69.9%) | 61 (79.2%) | 49 (68.1%) | 100 (65.8%) |
| Not reported | 64 (21.2%) | 10 (13.0%) | 15 (20.8%) | 39 (25.7%) |

Supplementary Table 2. PAT Items by Healthy People 2030 SDOH Domains, by Organ Group

|  | Combined Organ Groups  (*N=*302 caregivers) | Heart  (*n* = 77 caregivers) | Kidney  (*n* = 72 caregivers) | Liver  (*n* = 152 caregivers) |
| --- | --- | --- | --- | --- |
| **Education Access/Quality** |  |  |  |  |
| Caregiver did not complete high school | 9.9% | 9.1% | 6.9% | 8.6% |
| Patient not enrolled in school | 2.6% | 2.6% | 1.4% | 3.3% |
| **Healthcare Access/Quality** |  |  |  |  |
| On public insurance | 73.5% | 81.8% | 65.3% | 73.0% |
| Difficulty finding time for appointments | 8.0% | 6.5% | 8.3% | 8.6% |
| **Economic Stability** |  |  |  |  |
| Reported money problems | 34.1% | 33.8% | 36.1% | 33.6% |
| Difficulty meeting basic needs | 2.6% | 3.9% | -- | 3.3% |
| **Built Environment** |  |  |  |  |
| Rely on others for appointment transportation | 11.3% | 10.4% | 8.3% | 8.6% |
| 6+ people living in home | 15.3% | 9.1% | 12.5% | 19.1% |
| **Social/Community Context** |  |  |  |  |
| No family or community childcare support | 3.0% | 2.6% | 4.2% | 2.6% |
| Caregiver legal trouble or incarceration | 7.0% | 5.2% | 4.2% | 9.2% |
| Caregiver exposure to violence | 5.3% | 3.9% | 4.2% | 6.6% |
| Child exposure to violence | 3.3% | -- | 4.2% | 2.0% |
| Child custody concerns | 4.6% | 2.6% | 5.6% | 5.3% |
